# Evaluation of Polyphenol-Rich Lemon Peel Extract Use in a Zebrafish Model of Spinal Cord Injury: Morphology, Repair-Associated Markers, and Inflammatory Profile

**DOI:** 10.3390/ijms27031201

**Published:** 2026-01-25

**Authors:** Mirea Sicari, Lidia Pansera, Kamel Mhalhel, Marialuisa Aragona, Mariarosaria Galeano, Michele Rosario Colonna, Maria Levanti, Rosaria Laurà, Francesco Abbate, Antonino Germanà, Giuseppe Montalbano

**Affiliations:** 1 Zebrafish Neuromorphology Lab, Department of Veterinary Sciences, University of Messina, Polo Universitario dell’ Annunziata, 98168 Messina, Italy; mirea0497@gmail.com (M.S.); lipansera@unime.it (L.P.); mlaragona@unime.it (M.A.); mblevanti@unime.it (M.L.); laurar@unime.it (R.L.); abbatef@unime.it (F.A.); antonino.germana@unime.it (A.G.); 2 Department of Chemical, Biological, Pharmaceutical and Environmental Sciences, University of Messina, Viale Ferdinando Stagno D’Alcontres 31, 98166 Messina, Italy; 3 Department of Human Pathology and Evolutive Age “Gaetano Barresi”, University of Messina, Via C. Valeria, 98125 Messina, Italy; mariarosa.galeano@unime.it (M.G.); michelerosario.colonna@unime.it (M.R.C.)

**Keywords:** neuroregeneration, neuroprotection, natural extract, lemon peel extract, flavonoids, Zebrafish, spinal cord injury

## Abstract

Flavonoids are a diverse group of natural polyphenolic compounds, recognized for their ability to modulate cellular pathways and mitigate the pathological processes of many neurodegenerative diseases. This study investigates the neurotrophic potential of a polyphenolic-rich lemon peel extract (Lpe) in a Zebrafish larvae spinal cord injury (SCI) model. To evaluate its potential effects, embryos were divided into six experimental groups: a baseline control group in which larvae were neither subjected to spinal cord injury nor treated (Ctrl Group); a group with larvae subjected to spinal cord injury at 3 dpf without treatment (SCI Group); a group treated continuously with Lpe (25 µg/mL) from 0 to 5 dpf without injury (Continuous Group); a group treated continuously with Lpe and injured at 3 dpf (Continuous SCI Group); a group treated with Lpe starting at 3 dpf without injury (Curative Group); and finally, a group injured at 3 dpf and treated simultaneously with Lpe (Curative SCI Group). Lpe treatment significantly downregulated proinflammatory cytokines (*tnfa*, *il1b*, and *xcl8*), and modulated the neuroregenerative pathways Wnt/β catenin, and neurotrophic factor Bdnf. Immunohistochemical analysis further revealed Sox2-positive cells localized around the central canal, consistent with activation of ependymal progenitor populations involved in injury-induced repair processes. These findings support the exploration of Lpe for mitigating SCI-induced damage.

## 1. Introduction

The spinal cord is the most caudal segment of the central nervous system (CNS), an essential structure responsible for transmitting sensory inputs and motor commands between the brain and the peripheral targets [1,2]. In mammals, it is composed of various cell populations, including neurons and glial and ependymal cells. During embryonic development, the neuroepithelial cells lining the neural tube develop into the CNS [3]. As neurogenesis begins, these neuroepithelial cells acquire glial characteristics and transform into radial glia [4], which in turn act as neural progenitor cells and undergo asymmetric division to generate neurons and glia cells during embryonic development [5]. As the spinal cord matures, most radial glia progressively differentiate into astrocytes, while a small subset differentiate into ependymal cells that line the central canal throughout the organism’s lifespan [6]. This ependymal cell lining represents a conserved feature across all amniotes. Under physiological conditions, they retain a limited capacity for self-renewal; although, their proliferative potential is mostly restricted after birth, with only limited activity persisting into adulthood [7,8]. In Zebrafish, as in mammals, radial glial cells originate from early neuroepithelial progenitors during initial development and subsequently differentiate into neurons and glia [9,10,11]. A key difference from mammals, however, is that both neuroepithelial and radial glial cells persist into adulthood, and maintain their role as the primary neural stem cell (NSC) populations in the CNS throughout lifespan [12]. In the fully developed Zebrafish spinal cord, distinct populations of oligodendrocytes and ependymal cells are present. Various terms have been used in the literature to describe cells adjacent to the central canal, including radial glia, ependymal cells, ependymoglia, and ependymal radial glia (ERGs), reflecting their dual ependymal and radial glial characteristics [13,14,15]. Under physiological conditions, these cells exhibit quiescence, remaining non-proliferative but retaining the ability to re-enter the cell cycle, self-renew, and generate progeny upon stimulation [16], similarly to mammalian ependymal cells, and extend radial processes typical of glial cells [11]. In mammals, this quiescent ependymal cell population can become proliferative after spinal cord injury, thereby generating new progenitors [17]. In Zebrafish, astrocytes are considered to be absent, with ependymoglia fulfilling many of their functional roles instead [11,18]. However, more recent evidence by Chen et al. [19] has identified a previously uncharacterized glial population within the larval Zebrafish CNS, arising through the transformation of radial glial cells, with astrocyte-like features. These cells express typical astrocytic markers, such as the glutamate transporter and glutamine synthetase (GS), and closely associated with synapses [19]. Together, the persistence of neurogenic cell populations and their intrinsic regenerative capacity make Zebrafish an advantageous vertebrate model to investigate the dynamic cellular responses and molecular programs activated following neurological injuries. Spinal cord injury (SCI) is a permanent, chronic condition that leads to a wide range of sensorimotor and autonomic deficits. Despite advances in both standard-of-care and experimental therapeutic approaches, their efficacy in promoting neurological and functional recovery in severely disabled individuals remains limited [20]. The pathogenesis of SCI is a complex process comprising two distinct but interrelated phases. The primary injury involves direct mechanical damage to the spinal cord tissue, leading to demyelination and necrosis of neurons and axons. The subsequent secondary phase is characterized by a cascade of pathophysiological events triggered in response to the initial insult, including local hemorrhage, ischemia, edema, oxidative stress, and inflammatory responses [21]. Activation of innate immune responses following primary injury plays a critical role in the secondary phase, leading to an inflammatory response and contributing to further immune cell-mediated apoptosis [22]. Moreover, in mammals, chronic inflammation is associated with the formation of a glial scar composed of reactive astrocytes and pericytes that proliferate and migrate to the lesion site [23]. Although this scar formation helps limit inflammation and protects uninjured tissue near the injury site [24], it also impairs regeneration by creating a barrier to axonal regrowth and by producing an inhibitory extracellular matrix (ECM) through chondroitin sulfate proteoglycans (CSPGs) [25,26], damaging signals in the disrupted microenvironment [27,28]. Current molecular therapeutic strategies for SCI primarily aim to promote axonal regrowth and protect neurons from secondary cell death, largely through anti-inflammatory and neuroprotective mechanisms. Thus, evaluating the therapeutic approaches using adjuvants such as flavonoids [29,30], have been considered for the treatment of injured neural tissue [31,32]. Flavonoids are a large family of polyphenolic secondary metabolites which, thanks to their chemical properties, are able to modulate a variety of cellular pathways involved in key biological processes. As a result, they could alleviate several pathological conditions such as aging [33], cancer [34], cardiovascular diseases [35], inflammatory disorders [36], and degenerative diseases [37,38]. Their neuroprotective effects are mainly exerted by inhibiting neuroinflammatory pathways and reducing proinflammatory cytokines, including tumor necrosis factor TNF-α and interleukins IL-1β and IL-6 [39]. Moreover, flavonoids have also been shown to modulate the PI3K/Akt and MAPK pathways, as well as other kinase-dependent mechanisms that regulate neuronal apoptosis, thereby promoting neuronal survival and synaptic plasticity [40]. Among natural sources, Citrus fruits, especially lemon (*Citrus* × *limon*), represent one of the richest dietary reservoirs of bioactive flavonoids [41], which have been reported to modulate key molecular pathways involved in inflammation and oxidative stress. Such properties make flavonoids attractive candidates for promoting neural repair after spinal cord injury. Moreover, owing to its remarkable regenerative capacity and suitability for high-throughput screening [42], as well as the ability of their larval and adult forms to model spinal cord bleeding, inflammation, and apoptosis [11,43,44], Zebrafish offer an excellent model for investigating the cellular and molecular mechanisms underlying neural repair and identifying novel neuroregeneration-enhancing compounds [45]. In line with current knowledge, this study evaluates, for the first time, the in vivo effects of a previously characterized polyphenol-rich lemon peel extract (Lpe) in a Zebrafish larvae spinal cord injury (SCI) model. The extraction of green peel sample was performed according to Pagliari et al. [41]. Briefly, peels were dried at 40 °C to constant weight, finely powdered, and 250 mg aliquots were subjected to hydroalcoholic extraction with 5 mL of ethanol–water (50% *v*/*v*) through three sonication cycles (10 min each) at room temperature. The supernatants obtained after centrifugation (6000 rpm, 5 min) were pooled, the organic solvent was removed under reduced pressure, and the aqueous phase was frozen and lyophilized. Quantitative UHPLC–HRMS/MS data from the selected extract revealed the presence of several phenolic and flavonoid compounds, including lucenin 2, apigenin 6,8-di-C-hexoside, orientin, isoscopoletin, 3-hydroxycoumarin, rutin, apigenin-7-O-glucoside, eriocitrin, naringenin-7-glucoside, scopoletin, naringenin-7-O-rutinoside, coumarin, hesperidin, eriocytrol, luteolin, and hesperitin. Data obtained from morphological, behavioral, immunohistochemical, and molecular analyses were used to evaluate locomotor recovery, modulation of inflammation, and expression of neurogenesis markers following SCI; moreover, we sought to determine whether the timing of Lpe administration could modulate the therapeutic efficacy of the treatment.

## 2. Results

### 2.1. Swimming Performance Parameters by DanioVision Analysis

**Figure 1:**
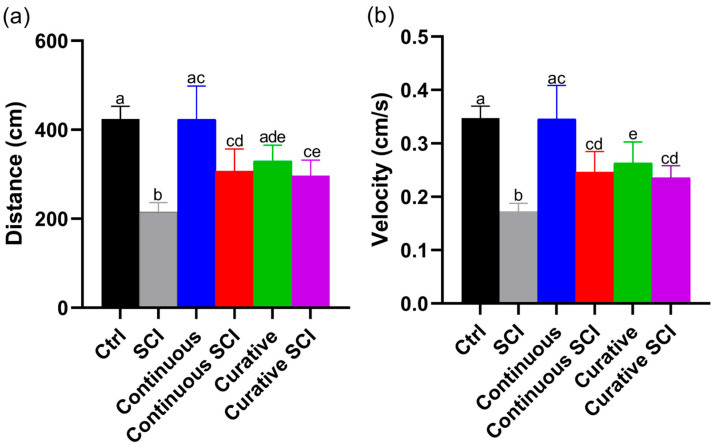
The locomotor behaviors of different experimental groups after exposure to Lpe (Continuous and Curative Groups), spinal cord injury (SCI Group), and the combined effect of the SCI and Lpe treatments (Continuous SCI Group, Curative SCI Group) at 5 days post fertilization. The Control (Ctrl) Group was maintained in E3 medium throughout the experiment. The larvae of SCI Group were kept in E3 medium and subjected to SCI at 72 hpf without Lpe treatment. The Continuous SCI Group was exposed to fresh Lpe from 0 to 120 hpf, covering both the pre- and post-injury periods. The Continuous Group followed the same treatment schedule without SCI. The Curative SCI Group remained in E3 medium until SCI was induced at 72 hpf, after which Lpe treatment continued until 120 hpf. The Curative Group was exposed to Lpe only after 72 hpf, without undergoing SCI. (**a**) Single-group cumulative swimming distance during the 120 min locomotor activity assay. (**b**) Mean locomotor speed during the 120 min assay under visible light. The data are expressed as the mean ± SD. Statistically significant differences, indicated by different letters (a, b, c, d, e) were determined by Welch’s ANOVA followed by Games–Howell post hoc tests at *p* < 0.05. Bars sharing at least one letter are not significantly different; bars with no letters in common differ significantly.

The locomotor activity of Zebrafish larvae, assessed as the mean of distance and swimming speed, was tracked during a 120 min free swim assay under visible light, revealed significant differences between the experimental groups, providing indications on the effectiveness of the various treatments. Even though the Continuous Group (Lpe administration without SCI from 0 to 120 hpf) showed comparable swimming performance to the Control (Ctrl) Group, the Curative Group (Lpe administration without SCI from 72 to 120 hpf) showed decreased, non-significant, motility compared to the Ctrl and the Continuous Groups. Moreover, the larvae in the Continuous SCI Group (Lpe administration from 0 to 120 hpf with SCI at 72 h) and the Curative SCI Group (SCI treated with Lpe from 72 to 120 hpf) showed statistically comparable traveled distance and swimming speed. Both parameters, however, were significantly increased compared to those in the SCI Group, although they were under the means of the Ctrl Group (Figure 1). In addition, no mortality was observed in any of the Lpe-treated groups in the days following spinal cord injury.

### 2.2. Accelerated Healing Across SCI Treatment Groups

**Figure 2:**
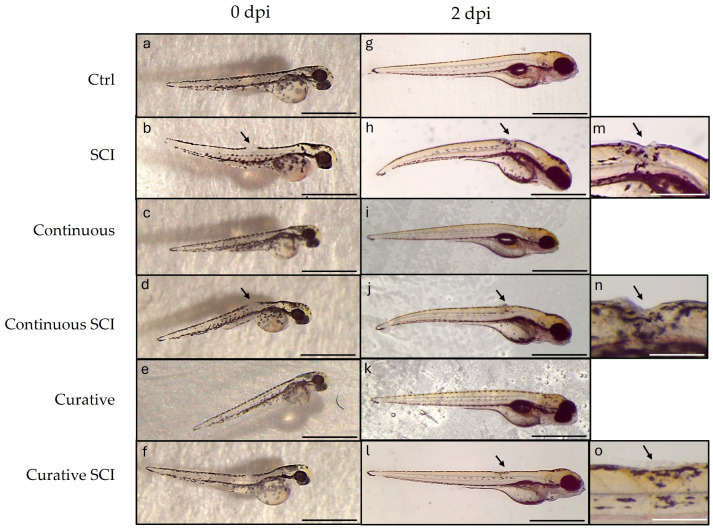
Representative images of spinal cord injury wound healing in Zebrafish larvae at 72 hpf (0 dpi) and 120 hpf (2 dpi) across the experimental groups: Ctrl (**a**,**g**), SCI (**b**,**h**,**m**), Continuous (**c**,**i**), Continuous SCI (**d**,**j**,**n**), Curative (**e**,**k**), and Curative SCI (**f**,**l**,**o**). The Control (Ctrl) Group was maintained in E3 medium throughout the experiment. The larvae of the SCI Group were kept in E3 medium and subjected to SCI at 72 hpf without Lpe treatment. The Continuous SCI Group was exposed to fresh Lpe from 0 to 120 hpf, covering both the pre- and post-injury periods. The Continuous Group followed the same treatment schedule without SCI. The Curative SCI Group remained in E3 medium until SCI was induced at 72 hpf, after which Lpe treatment continued until 120 hpf. The Curative Group was exposed to Lpe only after 72 hpf, without undergoing SCI. Arrows indicate the site of SCI. Magnification: (**a**–**l**) 2.5×; (**m**–**o**) 10×; (**m**–**o**) higher magnifications of (**h**,**i**,**k**), respectively. Scale bars: 1 mm (**a**–**l**) and 200 μm (**m**–**o**).

Following SCI, blood clotting occurs swiftly, effectively halting bleeding almost immediately after the injury induction (T0) (Figure 2a–f). Re-epithelialization began at 1 dpi, and healing was tracked until 2 dpi. The SCI Group showed minimal closure 2 h after SCI and partial healing by 2 dpi (Figure 2). The continuous SCI and Curative SCI Groups, however, showed notable healing by the second dpi. These findings were expressed within the linear mixed-effects model with group (SCI, Continuous, Curative), time (T0, 2 dpi), and their interaction as fixed effects of the lesion area. This highlighted a significant main effect of time (F(1,27) = 3914.94, *p* < 0.001), indicating a strong reduction in lesion area from T0 to 2 dpi. Moreover, the main effect of the group × time interaction was significant (F(2,27) = 4.20, *p* = 0.026), indicating that lesion evolution over time differed between experimental groups. The estimated marginal means indicated a greater reduction in lesion area in the Curative SCI Group compared with the Continuous SCI Group, which in turn showed better healing than the SCI larvae (see the Supplementary Materials).

### 2.3. Inflammatory Markers tnfa, il1b, and cxcl8 Profiling

**Figure 3:**
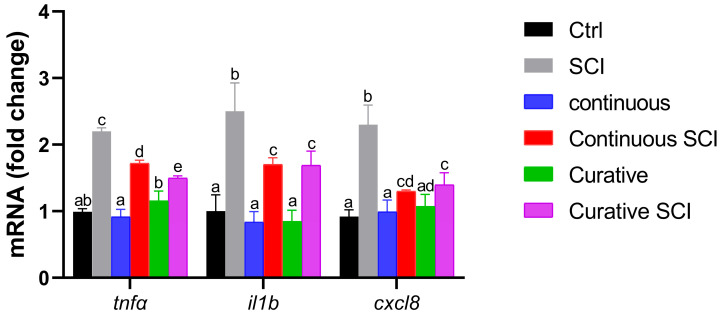
Inflammatory cytokines, *tnfa*, *il1b*, and *cxcl8* expression levels, among the different experimental groups: Ctrl, SCI, Continuous, Continuous SCI, Curative, and Curative SCI. The Control (Ctrl) Group was maintained in E3 medium throughout the experiment. The larvae of SCI Group was kept in E3 medium and subjected to SCI at 72 hpf without Lpe treatment. The Continuous SCI Group was exposed to fresh Lpe from 0 to 120 hpf, covering both the pre- and post-injury periods. The Continuous Group followed the same treatment schedule without SCI. The Curative SCI Group remained in E3 medium until SCI was induced at 72 hpf, after which Lpe treatment continued until 120 hpf. The Curative Group was exposed to Lpe only after 72 hpf, without undergoing SCI. Data are presented as mean ± SD. Statistically significant differences, indicated by different letters (a, b, c, d, e) were determined by Welch’s ANOVA followed by Games–Howell post hoc tests. Bars sharing at least one letter are not significantly different; bars with no letters in common differ significantly.

In order to assess the impact of Lpe on the inflammatory responses following SCI in Zebrafish larvae, the expression levels of proinflammatory cytokines (*tnfa*, *il1b*, and *cxcl8*) were assessed. The *tnfa* expression levels of those in the Continuous and Curative Groups were statistically different, while neither group differed significantly from the Control Group. Additionally, the SCI Group showed almost 2-fold the Ctrl *tnfa*; meanwhile, Lpe exposure, particularly in the SCI Curative Group, reduced *tnfa* expression significantly. The *il1b* in the Ctrl, Continuous, and Curative Groups had a comparable and statistically non-different expression level. The three groups’ expression levels represented half that of the SCI Group, while that of the Continuous SCI and Curative SCI Groups were significantly higher than that of Continuous and Curative SCI Groups, but still under that of the SCI Group. The same was true for *cxcl8*, where its expression levels in the Continuous and Curative Groups where comparable to that of the Ctrl Group. The results for both the Continuous SCI and Curative SCI Groups were comparable and statistically lower than those of the SCI Group (Figure 3).

### 2.4. Brain-Derived Neurotrophic Factor (Bdnf) Expression

**Figure 4:**
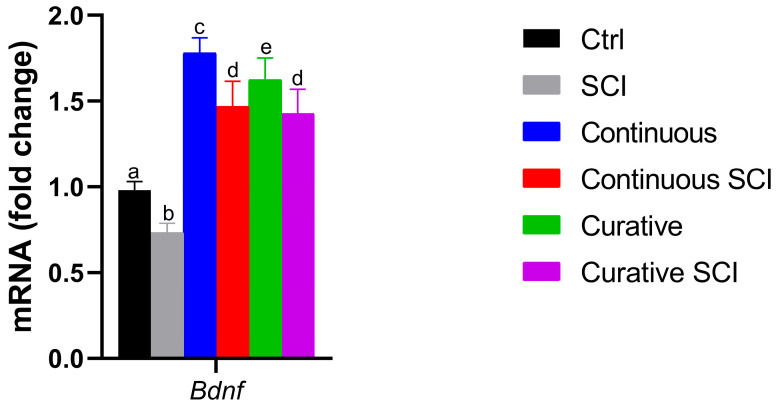
Bdnf expression levels in different experimental groups (Ctrl, SCI, Continuous, Continuous SCI, Curative, Curative SCI). The Control (Ctrl) Group was maintained in E3 medium throughout the experiment. The larvae of SCI Group were kept in E3 medium and subjected to SCI at 72 hpf without Lpe treatment. The Continuous SCI Group was exposed to fresh Lpe from 0 to 120 hpf, covering both the pre- and post-injury periods. The Continuous Group followed the same treatment schedule without SCI. The Curative SCI Group remained in E3 medium until SCI was induced at 72 hpf, after which Lpe treatment continued until 120 hpf. The Curative Group was exposed to Lpe only after 72 hpf, without undergoing SCI. Data are presented as mean ± SD. Statistically significant differences, indicated by different letters (a, b, c, d, e), were assessed by one way-ANOVA followed by Tukey post hoc test (*p* < 0.05). Bars sharing at least one letter are not significantly different; bars with no letters in common differ significantly.

To evaluate the Bdnf expression patterns after SCI, and its modulation by Lpe treatment, mRNA levels were analyzed across the experimental groups (Ctrl, SCI, Continuous, Continuous SCI, Curative, and Curative SCI). SCI showed the lowest Bdnf expression compared to the Ctrl Group and the treated groups, while the highest Bdnf expression level was reported in the Continuous and Curative Groups. Moreover, both the Continuous SCI and Curative SCI Groups showed a significant upregulation of Bdnf expression compared to the SCI Group, but these were still lower than that recorded in both the Lpe-treated groups without SCI (Figure 4).

### 2.5. Analysis of Wnt/β–Catenin-Related Gene Expression

**Figure 5:**
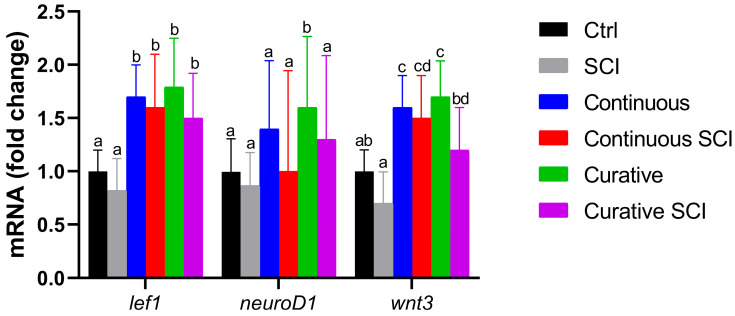
Wnt/β–catenin-related genes (lef1, Neurod1, and Wnt3) across different Zebrafish larvae experimental groups (Ctrl, Continuous, Continuous SCI, Curative, and Curative SCI). The Control (Ctrl) Group was maintained in E3 medium throughout the experiment. The larvae of the SCI Group were kept in E3 medium and subjected to SCI at 72 hpf without Lpe treatment. The Continuous SCI Group was exposed to fresh Lpe from 0 to 120 hpf, covering both the pre- and post-injury periods. The Continuous Group followed the same treatment schedule without SCI. The Curative SCI Group remained in E3 medium until SCI was induced at 72 hpf, after which Lpe treatment continued until 120 hpf. The Curative Group was exposed to Lpe only after 72 hpf, without undergoing SCI. Data are presented as mean ± SD. Statistically significant differences, indicated by different letters (a, b, c, and d), were assessed by one-way ANOVA followed by Turkey post hoc test (*p* < 0.05). Bars sharing at least one letter are not significantly different; bars with no letters in common differ significantly.

The Wnt/β–catenin signaling pathway was investigated through the analysis of mRNA expression levels of lef1, neurod1, and wnt3. These genes were analyzed in Zebrafish exposed to Lpe under different treatment conditions (Continuous, Continuous SCI, Curative, and Curative SCI) and compared with the Ctrl and SCI Groups (Figure 5).

**Figure 6:**
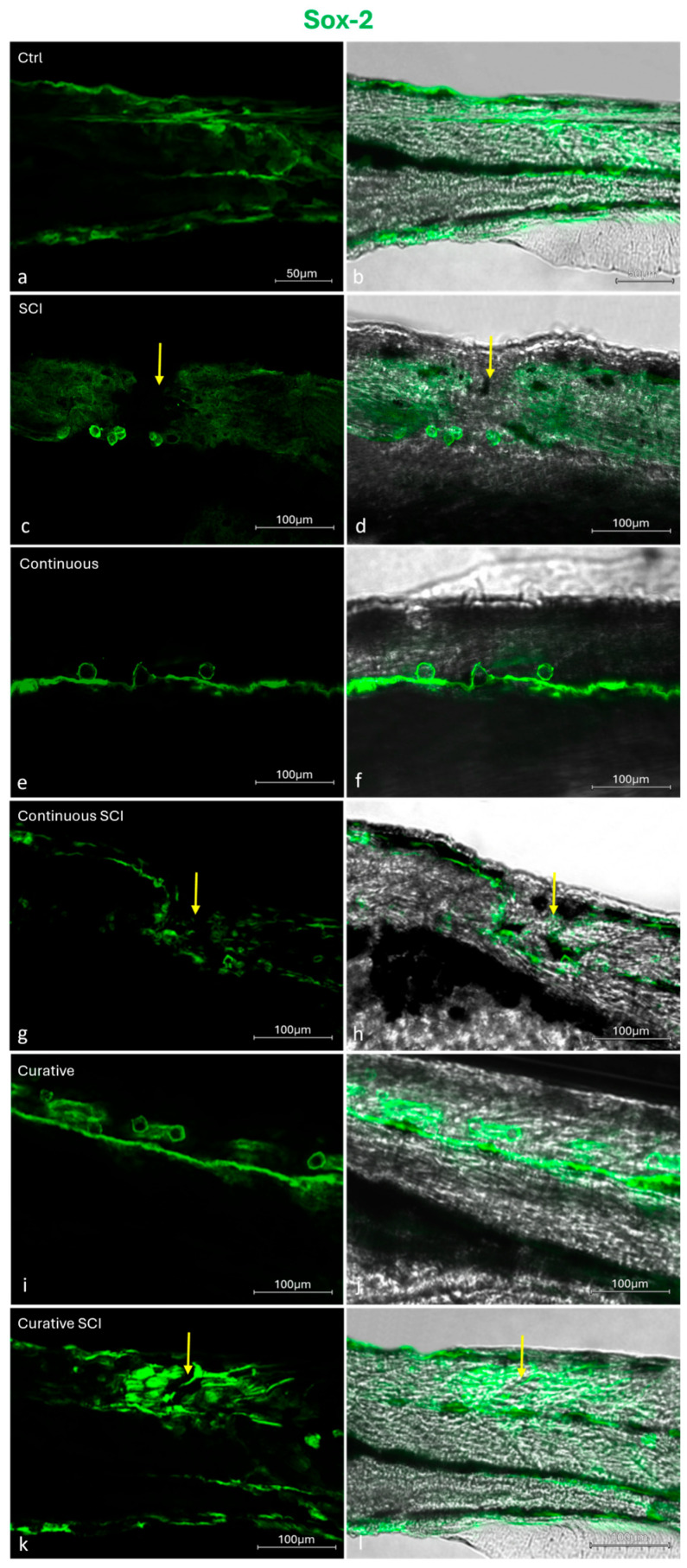
Sox2 immunolocalization across different Zebrafish larvae experimental groups. The Control (Ctrl) Group was maintained in E3 medium throughout the experiment. The larvae of the SCI Group were kept in E3 medium and subjected to SCI at 72 hpf without Lpe treatment. The Continuous SCI Group was exposed to fresh Lpe from 0 to 120 hpf, covering both the pre- and post-injury periods. The Continuous Group followed the same treatment schedule without SCI. The Curative SCI Group remained in E3 medium until SCI was induced at 72 hpf, after which Lpe treatment continued until 120 hpf. The Curative Group was exposed to Lpe only after 72 hpf, without undergoing SCI. (**a**) Ctrl Group and (**b**) transmitted view; (**c**) SCI Group and (**d**) transmitted view; (**e**) Continuous Group and (**f**) transmitted view; (**g**) Continuous SCI Group and (**h**) transmitted view; (**i**) Curative Group and (**j**) transmitted view; (**k**) Curative SCI Group and (**l**) transmitted view. The arrow indicates the site of spinal cord injury. Magnification 10×.

Immunofluorescence analysis revealed Sox2 immunopositivity across the experimental groups (Ctrl, Continuous, Continuous SCI, Curative, and Curative SCI) (Figure 6).

## 3. Discussion

Spinal cord injury (SCI) is one of the most severe forms of central nervous system trauma, leading to deep and often permanent impairments of motor, sensory, and autonomic functions. In front of the highly complex cascade of cellular and molecular responses triggered by SCI, a comprehensive investigation of both the underlying mechanisms of tissue damage and the endogenous regenerative processes [46,47,48] is essential for the development of effective and targeted therapeutic strategies. In this context, natural bioactive compounds have emerged as promising candidates for neurodegenerative therapy, given their potential to modulate inflammatory responses, promote neuroprotection, and support tissue regeneration, while typically exhibiting fewer side effects [47,48,49]. Among these, *Citrus limon* peel extract (Lpe) represents a particularly interesting source of polyphenolic compounds [50,51]. Within this framework, the present study investigated locomotor recovery, inflammatory responses, and molecular markers of neurogenesis following SCI in Zebrafish larvae, with particular emphasis on evaluating the potential modulatory effects of *Citrus limon* peel extract (Lpe) on these SCI-associated outcomes. To comprehensively evaluate the biological effects of Lpe, its chemical composition and safety profile were previously characterized [41], reveling 36 flavonoids, including luteolin, apigenin, rutin, naringenin, as well as coumarins and other polyphenols. Several of these compounds have already been characterized for their neuroprotective roles in CNS injury models. Among these, apigenin and luteolin suppress microglial activation and reduce proinflammatory cytokine production in vitro, pointing to direct anti-inflammatory mechanisms at the cellular level that may support neural protection [52]. In addition, naringenin, a flavonoid present in citrus fruits, demonstrated neuroprotective effects against chemically induced cerebellar damage in rats by attenuating oxidative stress and inflammatory markers such as TNF-α and IL-6, and by reducing apoptotic signaling [53]. Rutin has been reported to ameliorate neuroinflammation and secondary brain injury in a model of subarachnoid hemorrhage, likely through inhibition of RAGE–NF-κB mediated inflammatory pathways [54]. Other major constituents of lemon peel extracts, such as hesperidin and eriocitrin, also exhibit relevant bioactivities: hesperidin improves neuronal survival and reduces oxidative stress in animal models of neurotoxicity, partly via modulation of antioxidant enzymes and suppression of inflammatory mediators [55]; eriocitrin has been demonstrated to exert strong ROS-scavenging activity and to modulate Nrf2-related antioxidant pathways in vivo [56]. Integrating these data with our findings, the present study adds new in vivo evidence suggesting that the polyphenolic components of Lpe may also modulate additional pathways implicated in neural repair, including Bdnf and Wnt/β–catenin signaling during spinal cord injury, thereby offering preliminary insight into their potential combined contribution to early regenerative processes. In this context, the antioxidant potential of the Lpe extract was confirmed using spectrophotometric radical-scavenging assays (DPPH and ABTS) [41]. The locomotor activity in Zebrafish larvae was evaluated by assessing swimming distance and speed traveled during 120 min, revealing significant differences among the experimental groups and shedding light on the effectiveness of the various treatments. Whereas this study assessed locomotor performance under continuous light conditions [57], other experimental designs have evaluated similar parameters using light–dark cycles [58]. Larvae treated with Lpe either continuously or curatively but without SCI exhibited locomotor functions comparable to controls, proving the safety of the here used dose, chosen based on the LC50 of the same extract previously published [41]. The SCI Group showed a notable decline in motor function compared to the Ctrl Group. Although the groups in which SCI larvae were treated with Lpe showed a recovery of locomotor function comparable to that of the SCI Group, the statistical analysis demonstrated that there is no statistical significant differences between the Continuous SCI and Curative SCI experimental groups’ motility, highlighting a potential repair capacity guaranteed by the Lpe, and thus enhancing locomotor function in Zebrafish larvae. These findings are in line with those of previous studies, demonstrating that flavonoid-rich plant-derived extracts enhance both structural and functional healing after SCI in mammalian models [29,59,60]. Specifically, our study is accordance with the findings of a study proving citrus fruits’ naringin capacity to enhance motor recovery following SCI in rats [61]. In addition, numerous studies have linked *C. limon* flavonoids to cellular recovery stimulation, thereby highlighting the therapeutic potential of the *C. limon* polyphenolic extract in spinal cord regeneration [62,63,64].

Moreover, following SCI, wound healing was assessed under brightfield stereomicroscope. Blood clotting occurs swiftly, effectively halting bleeding almost immediately making 5 h post-wound an ideal time point to start observing and comparing wound healing, which was earlier to previous observations in adult Zebrafish models [59]. Re-epithelialization seems to begin at 1 dpi, and healing was tracked until 2 dpi. The SCI Group showed minimal closure 2 h after SCI and partial healing by 2 dpi, reflecting the physiological potential to regenerate in Zebrafish [65]. The Continuous SCI and Curative SCI Groups, however, showed notable healing by the second dpi, with the Curative SCI Group showing a greater reduction in the lesion area compared with the Continuous SCI Group, which in turn showed better healing than the SCI larvae. The current study findings highlight the potential of the Lpe-treatment to slightly faster early healing, with the curative treatment conferring balanced and rapid healing better than the continuous treatment in SCI larvae. The results revealed distinct responses among the experimental groups, underscoring the positive outcomes of Lpe treatments administered before and after SCI. The results highlight the well-documented role of flavonoids in promoting tissue repair and wound healing through multiple molecular pathways. Indeed, flavonoids are known to exert their regenerative effects by modulating signaling cascades such as Hedgehog, Nrf2/ARE, NF-κB, MAPK/ERK, Ras/Raf/MEK/ERK, and PI3K/Akt [66,67,68,69], crucial for regulating cellular proliferation, angiogenesis, inflammation, and oxidative stress, which collectively contribute to an improved microenvironment for tissue regeneration at the injury site [66]. The therapeutic potential of flavonoids is supported by a rising number of in vivo studies. For instance, hesperidin, a flavone glycoside, has been shown to significantly enhance wound healing in mammalian models, primarily by upregulating critical angiogenic and repair-related genes, including VEGF-c, Ang-1/Tie-2, TGF-β, and Smad-2/3 mRNA [70]. Similarly, rutin, demonstrated remarkable wound-healing activity modulating inflammatory mediators and promoting tissue remodeling [71]. The reported potential could be conferred to the capacity of the Lpe to improve the microenvironment surrounding the injury site, probably modulate inflammatory mediators and creating a more permissive environment for regeneration. For that, the inflammatory markers (*tnfa*, *il1b*, *cxcl8*) among the experimental groups were evaluated. Tumor necrosis factor-*tnfa* expression showed a differential response across treatment paradigms. Although the Continuous and Curative Groups treated with Lpe differed significantly from each other, neither group exhibited a statistically significant difference compared with the Ctrl Group, suggesting that Lpe exposure alone does not markedly alter basal *tnfa* expression; these results hint at the safety of the here-applied dose. The SCI Group exhibited the highest expression levels for all three genes, showing significant increases compared to the Ctrl Group, confirming the strong proinflammatory response induced by SCI. This upregulation was particularly expected as an aspect of the secondary injury cascade [72,73]. Moreover, elevated il-1β levels during late inflammation have been shown to impair regeneration, while its suppression helps axonal regeneration [68]. Similarly, in mammals, TNF signaling is rapidly upregulated after SCI and represents a central player in post-injury inflammation; its regulation is therefore considered a promising therapeutic strategy [69]. Notably, Lpe exposure attenuated this SCI-induced *tnfa* increase, with the most pronounced reduction observed in the Curative SCI Group. This finding indicates that Lpe may exert an anti-inflammatory effect, particularly when administered following injury, potentially limiting secondary inflammatory damage. Interleukin *il1b* and *cxcl8* expression levels in the Ctrl, Continuous, and Curative Groups were comparable and did not differ statistically, indicating that neither continuous nor curative Lpe exposure affected baseline *il1b* expression. However, in the Continuous SCI and Curative SCI Groups, both markers remained significantly higher than in their non-SCI counterparts. Still, their expression levels were lower than that observed in the SCI Group. The here-recorded significant reductions in the expressions of *tnfa*, *il1b*, and *cxcl8* were consistent with those reported in other studies: findings in which flavonoids derived from Citrus spp. have demonstrated neuroprotective effects [70,71,72]. This partial reduction suggests that Lpe mitigates, but does not completely suppress, the SCI-induced inflammatory response. The overall coincidences of the enhanced scarring of the wound, increased motility, in the SCI Groups treated by Lpe, could be explained by the reduced inflammation. Indeed, diverse studies on mammals have established a correlation between the reduced levels of proinflammatory cytokines and the mitigation of the secondary damage, limiting apoptosis, and enhancing neuronal survival and axonal regrowth [74,75]. The observed increase in proinflammatory cytokines following a spinal injury induces a neuroinflammatory environment that may interfere with brain-derived neurotrophic factor (Bdnf) function; simultaneously, Bdnf can modulate inflammation and promote regeneration [76]. The observed increase in proinflammatory cytokines following spinal cord injury (SCI prompted authors to consider potential neuroprotective pathways that may interfere with or modulate this inflammatory response. This led to the investigation of brain-derived neurotrophic factor (BDNF) and to profile its expression in the context of SCI and Lpe treatments, given its potential role promoting neural regeneration. Indeed, Bdnf, a key member of the neurotrophin family, plays an essential role in neuronal survival, development, and synaptic plasticity [77,78,79,80,81] and contributes to axonal regeneration and functional recovery in Zebrafish after traumatic injuries [82,83,84,85]. Research in SCI models indicates that elevated *Bdnf* levels are typically linked to improved functional outcomes, as Bdnf supports cell survival and tissue repair in both mammalian and teleost species [86,87]. The analysis of *Bdnf* expression revealed a distinct trend among the experimental groups. In the SCI Group, its expression level decreased significantly compared to that of the Ctrl Group. In contrast, the Continuous Group and, more notably, the Curative Group exhibited higher Bdnf levels than the Ctrl Group, suggesting that Lpe administration stimulates neurotrophic signaling and may enhance neuronal plasticity under physiological conditions. This upregulation is consistent with the reported ability of flavonoids to activate neuronal growth and synaptic maintenance pathways [88]. Following injury, the comparable Bdnf levels reported in the Continuous SCI and Curative SCI Groups were both higher than those of the Ctrl and SCI Groups but statistically lower than those of the Continuous and Curative Groups. Nonetheless, their expression levels remained lower than those observed in their uninjured counterparts. These findings are indicative of the *C. limon* extract’s neuroprotective and regenerative properties, suggesting its ability to promote neural repair by enhancing the expression of the neurotrophic factor Bdnf. Those findings are in accordance with the previous studies’ results reporting citrus flavonoids induced activation of ERK/MAPK signaling, a pathway known to induce the expression of *Bdnf* [89]. Another study conducted by Moghbelinejad et al. [90] assessed that rutin administration for rat Alzheimer models significantly increased the expression of key factors involved in cognitive function and neuronal survival, namely ERK1, CREB, and Bdnf, in the hippocampus. Additionally, luteolin, a citrus-derived flavonoid found at high concentrations in Lpe used in the current study, increases neurotrophic signaling and provides neuroprotection in models of ischemic damage by elevating *Bdnf* expression and reducing inflammatory responses [91]. Similarly, naringin fractioned in administrated Lpe has been associated with neuroprotection through the upregulation of *Bdnf* expression, aiding the regeneration of injured sciatic nerve in rats [61]. Neural repair is a complex, multifactorial process that also involves the activation of intracellular signaling cascades. Among these, the Wnt/β–catenin pathway has emerged as a critical regulator of neurogenesis, cell fate specification, and axonal regeneration after SCI. Beyond vertebrate regeneration, crosstalk between Bdnf and Wnt/β–catenin pathways has also been documented [92]. Thus, alongside assessing Bdnf expression, authors have profiled the potential of Lpe to modulate the Wnt/β–catenin pathway. For that, the expression of key genes associated with the aforementioned pathway (*lef1*, *neurod1*, and *wnt3*) was analyzed across the experimental groups, including the Ctrl Group, the SCI Group, and those treated with Lpe. The SCI Group showed a general decrease in the expression of all three genes compared to the Ctrl Group, although no statistical significance differences were reported. In contrast, both continuous and curative groups exhibited a clear upregulation of *wnt3* and *lef1* compared to the Ctrl and SCI Groups, with statistically significant differences. *Neurod1*, however, showed a milder, non-significant increase in the continuous treatment, while curative administration showed a higher expression compared to the Ctrl and SCI Groups. This pattern indicates that Lpe exposure has a potential modulating Wnt/β–catenin signaling pathway’ genes. Following injury, both the Continuous SCI and Curative SCI Groups maintained the higher expression levels of *wnt3* and *lef1* of the Continuous and Curative Groups, confirming that Lpe counteracts the injury-induced downregulation of these genes. However, *neurod1* expression remained unchanged, likely reflecting the temporal dynamics of its activation, as *neurod1* is typically upregulated during later stages of neuronal differentiation [93,94]. The Wnt/β–catenin signaling pathway plays a pivotal role in neuroregeneration following SCI, promoting neural stem cell proliferation and differentiation [95,96,97]. In Zebrafish, activation of Wnt/β–catenin signaling is required for radial glial neurogenesis and axonal regrowth after SCI [95,96]. Moreover, Wnt/β–catenin signaling controls the pro-regenerative extracellular matrix and supports axon navigation and functional recovery in Zebrafish SCI models [97]. These findings suggest that Lpe may modulate the Wnt/β–catenin signaling pathway, likely enhancing the regenerative potential of injured spinal tissue. Previous studies have highlighted the neuroregenerative effects of natural flavonoids on the Wnt/β–catenin pathway. Immunohistochemical analysis of Sox2, a transcription factor essential for maintaining neural progenitor identity and driving neurogenic reprogramming [98,99], in Zebrafish larvae SCI models revealed a immunolocalization of Sox2 in ependymal cells surrounding the central canal [14]. These Sox2+ cells should be identified as ependymal (or ependymo-radial) progenitor cells, based on established immunohistochemical studies showing Sox2 expression primarily in ependymal cells lining the central canal [14]. This pattern, mirrors canonical Zebrafish repair dynamics in which Sox2 orchestrates ependymal progenitor proliferation, gliogenesis, and neurogenesis to bridge lesioned tissue and restore locomotor function [100]. Taken together, these results support the involvement of bioactive compounds from Lpe in the modulation of intracellular signaling pathways following SCI, with particular emphasis on the regulation of key molecular mediators of neuronal plasticity and modulating inflammation.

Some limitations of this study should be acknowledged, including the use of a larval Zebrafish model, which may not fully recapitulate the complexity of SCI observed in adult vertebrates. Moreover, species-specific differences in regenerative responses should be considered when interpreting the results. Moreover, although Lpe cannot restore regeneration in higher vertebrates, their ability to modulate inflammation and influence BDNF, the Wnt/β–catenin pathway, and Sox2 signaling suggests that they may play a significant role in the formulation of dietary supplements which could be used alongside pharmacological therapies.

## 4. Materials and Methods

### 4.1. Citrus limon Peel Extracts: Characterization and Extraction

The *Citrus limon* peel extract (Lpe) used in the present study was selected following an analytical comparison performed across different lemon varieties, anatomical fruit sections (peel, albedo, pulp, and seeds), and ripening stages (green and yellow). The screening included the determination of total phenolic content (TPC) by the Folin–Ciocalteu method, antioxidant capacity assays (DPPH and ABTS), and a detailed ultrahigh-performance liquid chromatography–ultraviolet/high-resolution mass spectrometry/MS (UHPLC–UV/HRMS/MS) chemical profiling supported by multivariate analyses (PCA and PLS), as reported in Pagliari et al. [41].

These evaluations consistently identified the green peel as the sample with the highest phenolic enrichment, the strongest radical-scavenging activity, and the most distinct metabolomic signature, and it was therefore selected for the in vivo investigations.

The resulting dry extract was stored at −20 °C and freshly resuspended in E3 medium solution (E3 solution composition: 5 mM NaCl, 0.17 mM KCl, 0.33 mM CaCl_2_, 0.33 mM MgSO_4_) [101] at 25 μg/mL for the in vivo experiments.

### 4.2. Zebrafish Maintenance

Zebrafish AB (*Danio rerio*) were maintained at the Institute for Comparative, Experimental, Forensic, and Aquatic Pathology “Slavko Bambir”, Department of Chemical, Biological, Pharmaceutical, and Environmental Sciences of the University of Messina, Italy. The Zebrafish larvae from 0 to 120 hpf were maintained in a self-contained system (ZebTec, Tecniplast, Buguggiate, Varese, Italy) under controlled water conditions, with a 14 h light/10 h dark cycle, 27–28 °C, pH of 7.5, and 600 μS/cm conductivity. Fish were fed twice daily with Gemma micro 300 (Skretting, Varese, Italy) and Artemia salina, amounting to 3% of their body weight. After spawning, fertilized eggs were collected using a sieve, then non-fertilized eggs or damaged embryos were discarded.

### 4.3. Spinal Cord Injury

Three-day post fertilization (3 dpf) larvae were anesthetized in Ethyl 3-aminobenzoate methane sulfonate (MS-222; 0.01% final concentration), obtained by diluting 1 mL of a 0.4% stock solution in 40 mL of medium. Larvae remained in the anesthetic for 2–3 min until they no longer responded to gentle touch stimuli.

Once fully anesthetized, larvae were transferred to an agarose-coated Petri dish, which ensured stable immobilization during the surgical procedure, and placed in a lateral position. The spinal cord was fully transected, using the sharpest point of a syringe needle (30 G), at the level of the 15th myotome, carefully avoiding injury to the underlying notochord, as previously described [102,103]. The spinal cord transection was performed with a single smooth motion to ensure the lesion was V-shaped. After creating the lesion, each larva was transferred from the agar plate into a single well of a 24-well plate containing E3 medium without anesthetic to recover for 1 h. Then, depending on the experimental group, the larvae were placed in wells filled with the respective medium and maintained at 28.5 °C. On the same day of the surgery, dead or notochord-damaged larvae were removed, and the lesioned larvae were monitored daily. All procedures were performed under a stereomicroscope (Leica M205C equipped with a Leica IC80 HD digital camera, Leica, Milan, Italy).

### 4.4. Experimental Design and C. lemon Peel Extract Administration

**Figure 7:**
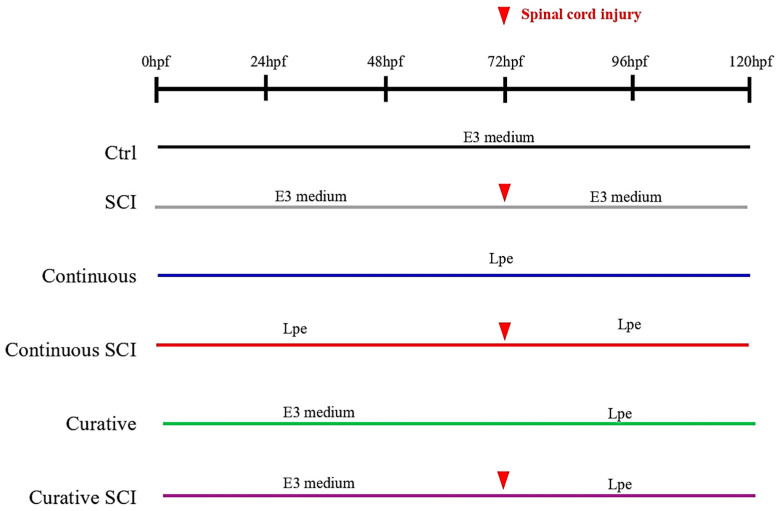
Experimental timeline showing the different treatment conditions in Zebrafish embryos, from 0 hpf to 120 hpf. Spinal cord injury (red arrowhead) was induced at 72 hpf.

The experimental design comprised larval groups (*n* = 20 per group, with three independent replicates), allowing comparison between spinal cord injury (SCI)-induced effects and those associated with *Citrus limon* peel extract (Lpe) treatment. The Control (Ctrl) Group, used as the uninjured baseline, was maintained in E3 medium throughout the experiment without exposure to SCI or Lpe. The SCI group served as the injury-only control; larvae were kept in E3 medium and subjected to SCI at 72 h post-fertilization (hpf) but did not receive Lpe treatment. In order to investigate the biological effects of Lpe at different timepoints, several treatment groups were included. The Continuous SCI Group was exposed to fresh Lpe (25 μg/mL in E3 medium) from 0 to 120 hpf, covering both the pre- and post-injury periods. The Continuous Group followed the same treatment schedule without SCI, allowing evaluation of Lpe effects in the absence of injury. The Curative SCI Group remained in E3 medium until SCI was induced at 72 hpf, after which Lpe treatment continued until 120 hpf. Similarly, the Curative Group was exposed to Lpe only after 72 hpf, without undergoing SCI, to assess the impact of delayed treatment in non-injured larvae. An overview of the experimental groups and treatment timelines is provided in Figure 7. Group allocation was performed by one operator prior to the start of the experiment. During the exposure phase, the operator conducting the treatments and daily maintenance was aware of group assignment to ensure correct handling of solutions. However, during outcome assessment the groups were identified solely by coded labels, and data analysis was carried out by a second researcher who remained blinded to the treatment group identities to minimize bias.

To minimize potential confounding factors, all experimental groups were maintained under identical environmental conditions and assessed at the same developmental stage and time points. The analyses were performed using the same experimental setup across groups. Moreover, all experimental groups were processed using a predefined order of plates during daily medium changes and subsequent experimental procedures.

### 4.5. Assessment of Functional Recovery (Danio Vision)

Larval motor function recovery was monitored using the DanioVision™ platform (Noldus, Wageningen, The Netherlands), according to the protocol detailed in (Mhalhel et al. [80]). At the experimental endpoint, at 120 hpf, larvae were transferred to individual wells of a 24-well transparent spot plate with 1 mL of E3 medium. Following a 10 min dark acclimation period, we assessed and documented behavioral changes of Zebrafish larvae using the DanioVisionTM observation system (Noldus, Wageningen, The Netherlands, Model: 17.0.1630) for 120 min. A behavioral assay was conducted in a temperature-controlled room at 26 ± 1 °C, and the light intensity was adjusted to 2412 lux. The accumulated behavioral activity data of each Zebrafish larva were analyzed across three endpoints. The total distance traveled and swimming velocity were quantified using Ethovision^®^XT (Noldus, VA, USA). After completing the behavioral analysis using the DanioVision™ system, larvae were examined and imaged with a Leica stereomicroscope (M205C) equipped with a Leica IC80 HD digital camera (Leica, Milan, Italy) to record the injury site and track morphological signs of recovery. The inherent optical transparency of Zebrafish larvae enables the high-resolution in vivo visualization of spinal cord regeneration, offering a dependable qualitative assessment of structural restoration over time.

### 4.6. Assessment of Wound Healing

Lesion area was quantified at T0 and 2 dpi for each of the ten larvae. Images were acquired using stereomicroscope (Leica M205C equipped with a Leica IC80 HD digital camera, Leica, Milan, Italy) under identical acquisition settings. The lesion area was measured using ImageJ/Fiji version 1.54 h (ImageJ/Fiji, Bethesda, MD, USA). Measurements were expressed in μm^2^.

### 4.7. RT-PCR

Gene expression analyses complied with the minimum information for publication of quantitative real-time PCR experiments, MIQE guidelines [84,85,104]. Total RNA was extracted from pools of 15 randomly selected larvae per experimental group using the QIAzol^®^ Lysis Reagent (QIAGEN^®^ Inc., Valencia, CA, USA, Cat. # 79306). The RNA was quantified using NanoDrop^®^ ND-1000 spectrophotometer (Thermo Fisher Scientific, Wilmington, DE, USA), and the quality verified running 500 ng of RNA on a 1% Agarose gel. Two micrograms of total RNA were reverse transcribed into cDNA using the QuantiTect^®^ Reverse Transcription Kit (QIAGEN Gmbh, Hilden, Germany, Cat. 205313). The analysis was carried out in triplicate, using the iTaq™ Universal SYBR^®^ Green Supermix (Bio-Rad Laboratories Inc., Hercules, CA, USA, Cat. #1725124). The primers were designed, using NCBI Primer-BLAST (https://www.ncbi.nlm.nih.gov/tools/primer-blast/, accessed on: 3 July 2025) [105] to bind the sequences of the here-studied genes. See Table 1 for the primer sequence details. Samples were amplified with the primers for each target gene, and for all these genes one no template control (NTC) sample was run. Raw data (CT) were analyzed with “Biogazelle qbase plus” software version 2.3, and the fold changes (versus starvation value) were expressed as CNRQ (calibrated normalized relative quantity) of the means with standard error (S.E.). β-actin and EF-1 alpha were used as the housekeeping genes. The starvation values are reported as 2^−ΔΔCT^.

### 4.8. Statistical Analysis

Statistical analyses were conducted, and graphs were created using IBM SPSS Statistics version 22 for Windows, (IBM Corp, Armonk, NY, USA) and GraphPad Prism version 8.0.1 for Windows (GraphPad Software, San Diego, CA USA) as described previously [106]. After confirming data normality and homogeneity of variance, the differences between the groups were analyzed using one-way ANOVA-based tests followed by Tukey’s or Games–Howell post hoc tests. Values are expressed as mean ± SE, and the significance threshold was established as the *p*-value (*p* < 0.05). Lesion area was analysed using a linear mixed-effects model with group (SCI, Continuous, Curative), time (T0, 2 dpi), and their interactions as the fixed effects. Time was specified as a repeated measure within subject, using an unstructured covariance matrix. The significance of the fixed effects was assessed using Type III tests. Estimated marginal means were calculated for each group × time condition. Results are reported as model-estimated means ± standard error, and a *p*-value < 0.05 was considered statistically significant.

### 4.9. Immunohistochemical Detection of Sox2

To analyze the immunolocalization of Sox2, larvae from different groups were fixed in 4% PFA in phosphate-buffered saline (PBS) (AAJ19943K2, Thermo Scientific, Waltham, MA, USA) 0.1 m (pH = 7.4), washed in phosphate-buffered saline (PBS), and processed for immunofluorescences technique. Larvae were washed in working buffer (Tris–HCl buffer 0.05 M, pH 7.5) containing 0.1% bovine serum albumin and 0.2% Triton-X 100 and incubated in 0.3% H_2_O_2_ (PBS) solution for 3 min to prevent the activity of endogenous peroxidase. Then, fetal bovine serum (F7524 Sigma-Aldrich, St. Louis, MO, USA) was added to the rinsed sections for 30 min to avoid non-specific binding. The incubation with Sox2 (GT1876) (MA5-31455, Molecular Probes, Invitrogen, Waltham, MA, USA) monoclonal antibody was carried out overnight at 4 °C in a humid chamber. Afterward, the incubated sections were washed in the working buffer and incubated for 1.5 h at room temperature with Goat anti-Mouse IgG (H+L) Cross-Adsorbed Secondary Antibody, Alexa Fluor™ 488 (A-11001, Molecular Probes, Invitrogen, Waltham, MA, USA). Finally, the sections were washed, dehydrated, and mounted with Fluoromount™ Aqueous Mounting Medium (Sigma Aldrich, Saint Louis, MO, USA). The sections were analyzed and images were acquired using a Zeiss LSMDUO confocal laser scanning microscope with a META module (Carl Zeiss MicroImaging GmbH, München, Germany) microscope (LSM700 AxioObserver Carl Zeiss MicroImaging GmbH, München, Germany). A Zen 2011 (LSM 700 Zeiss software ZEN 3.7 Carl Zeiss MicroImaging GmbH, München, Germany) built-in “transmitted view” was used. Each image was rapidly acquired to minimize photodegradation. To provide negative controls, representative sections were incubated with specifically preabsorbed antisera as described above. Under these conditions, no positive immunostaining was observed.

## 5. Conclusions

This study demonstrates that the polyphenolic-rich extract derived from the *Citrus limon* peel (Lpe) exerts significant benefits in a Zebrafish model of spinal cord injury (SCI). One of the most relevant outcomes of this research was the ability of Lpe to reduce inflammatory markers following SCI and activate crucial pathways such as Bdnf and Wnt/β–catenin, thereby promoting the modulation of post-injury microenvironment. In this context, the detection of Sox2-positive cells in the ependymal region supports the involvement of progenitor-related responses that are known to contribute to spinal cord repair in Zebrafish.

Collectively, these observations highlight Lpe as a promising source for complementary strategies aimed at limiting damage and promoting repair in SCI and, more broadly, in central nervous system trauma.

## Figures and Tables

**Table 1:** Primers for real-time PCR.

Gene	Primer Orientation	Nucleotide Sequence
*Beta-actin*	Forward	AAAGCTGTGACCCACCTCAC
	Reverse	CAGCTGGTCGTATCCGTTTG
*EF-1 alpha*	Forward	TGTCCTCAAGCCTGGTATGG
	Reverse	TGGGTCGTTCTTGCTGTCTC
*wnt3*	Forward	GTG GAA CTG CAC CAC TAT CA
	Reverse	GAG GCA ATC GCA TGA ACA AAT
*lef1*	Forward	CGG GAA GAG CAA GCT AAG TAT T
	Reverse	GCA GAC CAT CCT GGG TAA AG
*neurod1*	Forward	GAG GGT TAA GAG CAA GAC AGA C
	Reverse	CTG GGA GAT GCT TGA GGT TT
*Bdnf*	Forward	GCA CGG CAG AAG TTC TCA TA
	Reverse	CCT ATG CTG TCC TGG AGA TAG A
*cxcl8*	Forward	CTC TGC TCC ATG GGT TAA GAA G
	Reverse	AAC TTG ACT TCA CAG GTG ATC C
*il1b*	Forward	AGT GAA CGT GGT GGA TTC AG
	Reverse	TGC GCA CAG AGA CTT CTT ATA C
*tnfa*	Forward	CTT CCT CAG ACC ACG GAA A
	Reverse	CGA TTG TCC TGA AGG GTC A

## Data Availability

The original contributions presented in this study are included in the article/Supplementary Materials. Further inquiries can be directed to the corresponding authors.

## Supplementary Materials

The following supporting information can be downloaded at: https://www.mdpi.com/article/10.3390/ijms27031201/s1.
