# Supplementary material for: Evaluation of Polyphenol-Rich Lemon Peel Extract Use in a Zebrafish Model of Spinal Cord Injury: Morphology, Repair-Associated Markers, and Inflammatory Profile
Source: Int J Mol Sci. 2026 Jan 25;27(3):1201. doi: 10.3390/ijms27031201 (PMC12897618; doi:10.3390/ijms27031201)
Supplement: Supplementary file 1 [file ijms-27-01201-s001.zip › ijms-4087728-supplementary.pdf]

Supplementary Materials

Statistical Summary of Analyses for Figure 1

(a) Cumulative swimming distance during the 120-min locomotor activity assay.

ANOVA

		Sum of Squares	df	Mean Square	F	Sig.
Distance	Between Groups	282728,476	7	40389,782	10,639	,000
	Within Groups	189818,778	50	3796,376		
	Total	472547,254	57			

Robust Tests of Equality of Means

		Statistic[a]	df1	df2	Sig.
Distance	Welch	25,656	7	17,034	,000

a. Asymptotically F distributed.

Post Hoc Tests

Multiple Comparisons

Games-Howell

Dependent Variable	(I) Group	(J) Group	Mean Difference (I-J)	Std. Error	Sig.	95% Confidence Interval Lower Bound	95% Confidence Interval Upper Bound
Distance (Cm)	Ctrl	SCI	209,4224000*	15,3758925	,000	147,059581	271,785219
		Continuous	1,0770000	35,8043129	1,000	-163,774664	165,928664
		Continuous SCI	117,4958286*	22,6910177	,007	31,814287	203,177371
		Curative	94,5766500	21,8708933	,054	-1,811693	190,964993
		Curative SCI	126,9074000*	18,8749424	,001	54,405222	199,409578
	SCI	Ctrl	-209,4224000*	15,3758925	,000	-271,785219	-147,059581

	Continuous	-208,3454000*	34,5828164	,022	-376,630807	-40,059993
	Continuous SCI	-91,9265714*	20,7100133	,025	-172,913729	-10,939414
	Curative	-114,8457500*	19,8080373	,027	-212,254585	-17,436915
	Curative SCI	-82,5150000*	16,4403716	,012	-146,829788	-18,200212
Continuous	Ctrl	-1,0770000	35,8043129	1,000	-165,928664	163,774664
	SCI	208,3454000*	34,5828164	,022	40,059993	376,630807
	Continuous SCI	116,4188286	38,3983776	,185	-45,331077	278,168734
	Curative	93,4996500	37,9195081	,351	-71,165388	258,164688
	Curative SCI	125,8304000	36,2741860	,134	-37,848171	289,508971
Continuous SCI	Ctrl	-117,4958286*	22,6910177	,007	-203,177371	-31,814287
	SCI	91,9265714*	20,7100133	,025	10,939414	172,913729
	Continuous	-116,4188286	38,3983776	,185	-278,168734	45,331077
	Curative	-22,9191786	25,9002423	,980	-124,495800	78,657443
	Curative SCI	9,4115714	23,4254142	1,000	-77,423501	96,246644

Curative	Ctrl	-94,5766500	21,8708933	,054	-190,964993	1,811693
	SCI	114,8457500*	19,8080373	,027	17,436915	212,254585
	Continuous	-93,4996500	37,9195081	,351	-258,164688	71,165388
	Continuous SCI	22,9191786	25,9002423	,980	-78,657443	124,495800
	Curative SCI	32,3307500	22,6319182	,820	-63,574088	128,235588
Curative SCI	Ctrl	-126,9074000*	18,8749424	,001	-199,409578	-54,405222
	SCI	82,5150000*	16,4403716	,012	18,200212	146,829788
	Continuous	-125,8304000	36,2741860	,134	-289,508971	37,848171
	Continuous SCI	-9,4115714	23,4254142	1,000	-96,246644	77,423501
	Curative	-32,3307500	22,6319182	,820	-128,235588	63,574088

*. The mean difference is significant at the 0.05 level.

(b) Mean locomotor speed during the 120-min assay under visible light.

Oneway

ANOVA

		Sum of Squares	df	Mean Square	F	Sig.
Velocity	Between Groups	,330	7	,047	18,066	,000
	Within Groups	,133	51	,003		
	Total	,463	58			

Robust Tests of Equality of Means

	Statistic[a]	df1	df2	Sig.

Velocity	Welch	46,662	7	17,489	,000

a. Asymptotically F distributed.

Post Hoc Tests

Multiple Comparisons

Games-Howell

Dependent Variable	(I) Group	(J) Group	Mean Difference (I-J)	Std. Error	Sig.	95% Confidence Interval Lower Bound	95% Confidence Interval Upper Bound
Velocity	Ctrl	SCI	,1636935*	,0124398	,000	,113991	,213396
		Continuous	,0013562	,0301745	1,000	-,139029	,141741
		Continuous SCI	,1009632*	,0178558	,004	,033640	,168287
		Curative	,2884477*	,0156997	,000	,221952	,354943
		Curative SCI	,1114900*	,0136928	,000	,058331	,164649
	SCI	Ctrl	-,1636935*	,0124398	,000	-,213396	-,113991
		Continuous	-,1623373*	,0293106	,031	-,305311	-,019364
		Continuous SCI	-,0627303	,0163537	,052	-,126029	,000569
		Curative	,1247542*	,0139676	,002	,060893	,188615
		Curative SCI	-,0522035*	,0116665	,018	-,096053	-,008354
	Continuous	Ctrl	-,0013562	,0301745	1,000	-,141741	,139029
		SCI	,1623373*	,0293106	,031	,019364	,305311
		Continuous SCI	,0996070	,0319874	,175	-,037801	,237015
		Curative	,2870915*	,0308358	,002	,147318	,426865
		Curative SCI	,1101338	,0298640	,121	-,030915	,251182

Continuous SCI	Ctrl	-,1009632*	,0178558	,004	-,168287	-,033640
	SCI	,0627303	,0163537	,052	-,000569	,126029
	Continuous	-,0996070	,0319874	,175	-,237015	,037801
	Curative	,1874845*	,0189519	,000	,114351	,260618
	Curative SCI	,0105267	,0173260	,998	-,054725	,075778
Curative	Ctrl	-,2884477*	,0156997	,000	-,354943	-,221952
	SCI	-,1247542*	,0139676	,002	-,188615	-,060893
	Continuous	-,2870915*	,0308358	,002	-,426865	-,147318
	Continuous SCI	-,1874845*	,0189519	,000	-,260618	-,114351
	Curative SCI	-,1769578*	,0150943	,000	-,241301	-,112615
Curative SCI	Ctrl	-,1114900*	,0136928	,000	-,164649	-,058331
	SCI	,0522035*	,0116665	,018	,008354	,096053
	Continuous	-,1101338	,0298640	,121	-,251182	,030915
	Continuous SCI	-,0105267	,0173260	,998	-,075778	,054725
	Curative	,1769578*	,0150943	,000	,112615	,241301

*. The mean difference is significant at the 0.05 level.

Assessment of Wound healing (Linear mixed-effects model)

Fixed Effects

Type III Tests of Fixed Effects[a]

Source	Numerator df	Denominator df	F	Sig.
Intercept	1	27,000	55777,431	,000
Group	2	27,000	96,870	,000
Time	1	27,000	3914,941	,000
Group * Time	2	27,000	4,198	,026

a. Dependent Variable: Lesion_area.

Estimated Marginal Means

Group * Time[a]

					95% Confidence Interval	
Group	Time	Mean	Std. Error	df	Lower Bound	Upper Bound
SCI	T0	18928,014	167,153	27,000	18585,045	19270,984
	2Dpi	8559,498	187,534	27,000	8174,711	8944,286
Curative SCI	T0	18141,564	167,153	27,000	17798,595	18484,534
	2Dpi	6547,499	187,534	27,000	6162,711	6932,286
Continous SCI	T0	17428,014	167,153	27,000	17085,045	17770,984
	2Dpi	6607,498	187,534	27,000	6222,711	6992,286

a. Dependent Variable: Lesion_area.

Statistical Summary of Analyses for Figure 3

tnf-α expression levels, among the different experimental groups

ANOVA

TNFα

	Sum of Squares	df	Mean Square	F	Sig.
Between Groups	4,805	5	,961	527,931	,000
Within Groups	,033	18	,002		
Total	4,837	23			

Robust Tests of Equality of Means

TNFα

	Statistic[a]	df1	df2	Sig.
Welch	788,894	5	8,241	,000

a. Asymptotically F distributed.

Post Hoc Tests

Multiple Comparisons

Dependent Variable: TNFα

Games-Howell

(I) Group	(J) Group	Mean Difference (I-J)	Std. Error	Sig.	95% Confidence Interval	
					Lower Bound	Upper Bound
Ctrl	SCI	-1,2000000*	,0181978	,000	-1,272653	-1,127347
	Continus	,0797250	,0297392	,252	-,058196	,217646
	Continus SCI	-,7200500*	,0170538	,000	-,787943	-,652157
	Curative	-,1549250	,0399040	,107	-,355255	,045405
	Curative SCI	-,5002500*	,0151857	,000	-,562583	-,437917
SCI	Ctrl	1,2000000*	,0181978	,000	1,127347	1,272653
	Continus	1,2797250*	,0302662	,000	1,142919	1,416531
	Continus SCI	,4799500*	,0179570	,000	,408097	,551803
	Curative	1,0450750*	,0402982	,000	,846799	1,243351
	Curative SCI	,6997500*	,0161934	,000	,631935	,767565
Continus	Ctrl	-,0797250	,0297392	,252	-,217646	,058196
	SCI	-1,2797250*	,0302662	,000	-1,416531	-1,142919
	Continus SCI	-,7997750*	,0295925	,000	-,938098	-,661452
	Curative	-,2346500*	,0466606	,022	-,427270	-,042030
	Curative SCI	-,5799750*	,0285567	,000	-,722497	-,437453
Continus SCI	Ctrl	,7200500*	,0170538	,000	,652157	,787943
	SCI	-,4799500*	,0179570	,000	-,551803	-,408097
	Continus	,7997750*	,0295925	,000	,661452	,938098
	Curative	,5651250*	,0397947	,002	,364169	,766081
	Curative SCI	,2198000*	,0148963	,000	,158986	,280614
Curative	Ctrl	,1549250	,0399040	,107	-,045405	,355255
	SCI	-1,0450750*	,0402982	,000	-1,243351	-,846799
	Continus	,2346500*	,0466606	,022	,042030	,427270
	Continus SCI	-,5651250*	,0397947	,002	-,766081	-,364169
	Curative SCI	-,3453250*	,0390307	,010	-,551432	-,139218
Curative SCI	Ctrl	,5002500*	,0151857	,000	,437917	,562583
	SCI	-,6997500*	,0161934	,000	-,767565	-,631935
	Continus	,5799750*	,0285567	,000	,437453	,722497
	Continus SCI	-,2198000*	,0148963	,000	-,280614	-,158986
	Curative	,3453250*	,0390307	,010	,139218	,551432

*. The mean difference is significant at the 0.05 level.

il-1β expression levels, among the different experimental groups

ANOVA

IL1β

	Sum of Squares	df	Mean Square	F	Sig.
Between Groups	8,636	5	1,727	117,082	,000
Within Groups	,266	18	,015		
Total	8,902	23			

Robust Tests of Equality of Means

IL1β

	Statistic[a]	df1	df2	Sig.
Welch	104,051	5	8,185	,000

a. Asymptotically F distributed.

Post Hoc Tests

Multiple Comparisons

Dependent Variable: IL1β

Games-Howell

(I) Group	(J) Group	Mean Difference (I-J)	Std. Error	Sig.	95% Confidence Interval	
					Lower Bound	Upper Bound
Ctrl	SCI	-1,5000000*	,1234234	,001	-2,037257	-,962743
	Continus	,1625000	,0729583	,358	-,145200	,470200
	Continus SCI	-,7025000*	,0663796	,003	-1,016497	-,388503
	Curative	,1500000	,0738241	,430	-,158567	,458567
	Curative SCI	-,6900000*	,0819553	,001	-1,017703	-,362297
SCI	Ctrl	1,5000000*	,1234234	,001	,962743	2,037257
	Continus	1,6625000*	,1142639	,001	1,105699	2,219301
	Continus SCI	,7975000*	,1101798	,019	,216430	1,378570
	Curative	1,6500000*	,1148187	,001	1,095568	2,204432
	Curative SCI	,8100000*	,1202082	,011	,269975	1,350025
Continus	Ctrl	-,1625000	,0729583	,358	-,470200	,145200
	SCI	-1,6625000*	,1142639	,001	-2,219301	-1,105699
	Continus SCI	-,8650000*	,0472141	,000	-1,064250	-,665750
	Curative	-,0125000	,0572094	1,000	-,240303	,215303
	Curative SCI	-,8525000*	,0673764	,000	-1,129534	-,575466
Continus SCI	Ctrl	,7025000*	,0663796	,003	,388503	1,016497
	SCI	-,7975000*	,1101798	,019	-1,378570	-,216430
	Continus	,8650000*	,0472141	,000	,665750	1,064250
	Curative	,8525000*	,0485412	,000	,645701	1,059299
	Curative SCI	,0125000	,0601907	1,000	-,263629	,288629
Curative	Ctrl	-,1500000	,0738241	,430	-,458567	,158567
	SCI	-1,6500000*	,1148187	,001	-2,204432	-1,095568

	Continus	,0125000	,0572094	1,000	-,215303	,240303
	Continus SCI	-,8525000*	,0485412	,000	-1,059299	-,645701
	Curative SCI	-,8400000*	,0683130	,000	-1,118887	-,561113
Curative SCI	Ctrl	,6900000*	,0819553	,001	,362297	1,017703
	SCI	-,8100000*	,1202082	,011	-1,350025	-,269975
	Continus	,8525000*	,0673764	,000	,575466	1,129534
	Continus SCI	-,0125000	,0601907	1,000	-,288629	,263629
	Curative	,8400000*	,0683130	,000	,561113	1,118887

*. The mean difference is significant at the 0.05 level.

il-8 expression levels, among the different experimental groups

ANOVA

Il8

	Sum of Squares	df	Mean Square	F	Sig.
Between Groups	5,197	5	1,039	120,477	,000
Within Groups	,155	18	,009		
Total	5,353	23			

Robust Tests of Equality of Means

Il8

	Statistic[a]	df1	df2	Sig.
Welch	64,838	5	7,423	,000

a. Asymptotically F distributed.

Post Hoc Tests

Multiple Comparisons

Dependent Variable: Il8

Games-Howell

(I) Group	(J) Group	Mean Difference (I-J)	Std. Error	Sig.	95% Confidence Interval Lower Bound	95% Confidence Interval Upper Bound
Ctrl	SCI	-1,38152*	,07857	,001	-1,7673	-,9958
	Continus	-,07402	,05559	,762	-,3194	,1714
	Continus SCI	-,37902*	,02767	,002	-,5196	-,2384
	Curative	-,14902	,05468	,228	-,3891	,0910
	Curative SCI	-,48402*	,05224	,002	-,7097	-,2583
SCI	Ctrl	1,38152*	,07857	,001	,9958	1,7673
	Continus	1,30750*	,08866	,000	,9353	1,6797
	Continus SCI	1,00250*	,07440	,004	,5867	1,4183
	Curative	1,23250*	,08809	,000	,8609	1,6041
	Curative SCI	,89750*	,08660	,001	,5267	1,2683
Continus	Ctrl	,07402	,05559	,762	-,1714	,3194

	SCI	-1,30750*	,08866	,000	-1,6797	-,9353
	Continus SCI	-,30500*	,04952	,036	-,5761	-,0339
	Curative	-,07500	,06839	,867	-,3472	,1972
	Curative SCI	-,41000*	,06646	,006	-,6751	-,1449
Continus SCI	Ctrl	,37902*	,02767	,002	,2384	,5196
	SCI	-1,00250*	,07440	,004	-1,4183	-,5867
	Continus	,30500*	,04952	,036	,0339	,5761
	Curative	,23000	,04851	,074	-,0352	,4952
	Curative SCI	-,10500	,04574	,387	-,3539	,1439
Curative	Ctrl	,14902	,05468	,228	-,0910	,3891
	SCI	-1,23250*	,08809	,000	-1,6041	-,8609
	Continus	,07500	,06839	,867	-,1972	,3472
	Continus SCI	-,23000	,04851	,074	-,4952	,0352
	Curative SCI	-,33500*	,06570	,016	-,5968	-,0732
Curative SCI	Ctrl	,48402*	,05224	,002	,2583	,7097
	SCI	-,89750*	,08660	,001	-1,2683	-,5267
	Continus	,41000*	,06646	,006	,1449	,6751
	Continus SCI	,10500	,04574	,387	-,1439	,3539
	Curative	,33500*	,06570	,016	,0732	,5968

*. The mean difference is significant at the 0.05 level.

Statistical Summary of Analyses for Figure 4

ANOVA

BDNF

	Sum of Squares	df	Mean Square	F	Sig.
Between Groups	3,194	5	,639	215,647	,000
Within Groups	,053	18	,003		
Total	3,247	23			

Post Hoc Tests

Multiple Comparisons

Dependent Variable: BDNF

Tukey HSD

(I) Group	(J) Group	Mean Difference (I-J)	Std. Error	Sig.	95% Confidence Interval Lower Bound	Upper Bound
Ctrl	SCI	,24877*	,03849	,000	,1265	,3711
	Continus	-,80026*	,03849	,000	-,9226	-,6779
	Continus SCI	-,49026*	,03849	,000	-,6126	-,3679
	Curative	-,64326*	,03849	,000	-,7656	-,5209
	Curative SCI	-,44608*	,03849	,000	-,5684	-,3238
SCI	Ctrl	-,24877*	,03849	,000	-,3711	-,1265

	Continus	-1,04902*	,03849	,000	-1,1713	-,9267
	Continus SCI	-,73903*	,03849	,000	-,8613	-,6167
	Curative	-,89203*	,03849	,000	-1,0143	-,7697
	Curative SCI	-,69485*	,03849	,000	-,8172	-,5725
Continus	Ctrl	,80026*	,03849	,000	,6779	,9226
	SCI	1,04902*	,03849	,000	,9267	1,1713
	Continus SCI	,31000*	,03849	,000	,1877	,4323
	Curative	,15700*	,03849	,008	,0347	,2793
	Curative SCI	,35417*	,03849	,000	,2319	,4765
Continus SCI	Ctrl	,49026*	,03849	,000	,3679	,6126
	SCI	,73903*	,03849	,000	,6167	,8613
	Continus	-,31000*	,03849	,000	-,4323	-,1877
	Curative	-,15300*	,03849	,010	-,2753	-,0307
	Curative SCI	,04418	,03849	,855	-,0781	,1665
Curative	Ctrl	,64326*	,03849	,000	,5209	,7656
	SCI	,89203*	,03849	,000	,7697	1,0143
	Continus	-,15700*	,03849	,008	-,2793	-,0347
	Continus SCI	,15300*	,03849	,010	,0307	,2753
	Curative SCI	,19718*	,03849	,001	,0749	,3195
Curative SCI	Ctrl	,44608*	,03849	,000	,3238	,5684
	SCI	,69485*	,03849	,000	,5725	,8172
	Continus	-,35417*	,03849	,000	-,4765	-,2319
	Continus SCI	-,04418	,03849	,855	-,1665	,0781
	Curative	-,19718*	,03849	,001	-,3195	-,0749

*. The mean difference is significant at the 0.05 level.

Statistical Summary of Analyses for Figure 5

(a) *lef1 expression levels across different zebrafish larvae experimental groups*

ANOVA

lef1

	Sum of Squares	df	Mean Square	F	Sig.
Between Groups	3,185	5	,637	17,828	,000
Within Groups	,643	18	,036		
Total	3,828	23			

Post Hoc Tests

Multiple Comparisons

Dependent Variable: lef1

Tukey HSD

(I) Group	(J) Group	Mean Difference (I-J)	Std. Error	Sig.	95% Confidence Interval	
					Lower Bound	Upper Bound
Ctrl	SCI	,180300	,133650	,755	-,24444	,60504
	Continus	-,699700*	,133650	,001	-1,12444	-,27496
	Continus SCI	-,599700*	,133650	,003	-1,02444	-,17496
	Curative	-,799700*	,133650	,000	-1,22444	-,37496
	Curative SCI	-,499950*	,133650	,016	-,92469	-,07521
SCI	Ctrl	-,180300	,133650	,755	-,60504	,24444
	Continus	-,880000*	,133650	,000	-1,30474	-,45526
	Continus SCI	-,780000*	,133650	,000	-1,20474	-,35526
	Curative	-,980000*	,133650	,000	-1,40474	-,55526
	Curative SCI	-,680250*	,133650	,001	-1,10499	-,25551
Continus	Ctrl	,699700*	,133650	,001	,27496	1,12444
	SCI	,880000*	,133650	,000	,45526	1,30474
	Continus SCI	,100000	,133650	,973	-,32474	,52474
	Curative	-,100000	,133650	,973	-,52474	,32474
	Curative SCI	,199750	,133650	,672	-,22499	,62449
Continus SCI	Ctrl	,599700*	,133650	,003	,17496	1,02444
	SCI	,780000*	,133650	,000	,35526	1,20474
	Continus	-,100000	,133650	,973	-,52474	,32474
	Curative	-,200000	,133650	,671	-,62474	,22474
	Curative SCI	,099750	,133650	,973	-,32499	,52449
Curative	Ctrl	,799700*	,133650	,000	,37496	1,22444
	SCI	,980000*	,133650	,000	,55526	1,40474
	Continus	,100000	,133650	,973	-,32474	,52474
	Continus SCI	,200000	,133650	,671	-,22474	,62474
	Curative SCI	,299750	,133650	,267	-,12499	,72449
Curative SCI	Ctrl	,499950*	,133650	,016	,07521	,92469
	SCI	,680250*	,133650	,001	,25551	1,10499
	Continus	-,199750	,133650	,672	-,62449	,22499
	Continus SCI	-,099750	,133650	,973	-,52449	,32499
	Curative	-,299750	,133650	,267	-,72449	,12499

*. The mean difference is significant at the 0.05 level.

(b) Neurod1 expression levels across different zebrafish larvae experimental groups

ANOVA

NeuroD

	Sum of Squares	df	Mean Square	F	Sig.
Between Groups	1,612	5	,322	7,517	,001
Within Groups	,772	18	,043		

Total	2,384	23			

Post Hoc Tests

Multiple Comparisons

Dependent Variable: NeuroD

Tukey HSD

(I) Group	(J) Group	Mean Difference (I-J)	Std. Error	Sig.	95% Confidence Interval Lower Bound	Upper Bound
Ctrl	SCI	,130000	,146427	,945	-,33535	,59535
	Continus	-,402750	,146427	,113	-,86810	,06260
	Continus SCI	-,004500	,146427	1,000	-,46985	,46085
	Curative	-,604750*	,146427	,007	-1,07010	-,13940
	Curative SCI	-,304500	,146427	,340	-,76985	,16085
SCI	Ctrl	-,130000	,146427	,945	-,59535	,33535
	Continus	-,532750*	,146427	,020	-,99810	-,06740
	Continus SCI	-,134500	,146427	,937	-,59985	,33085
	Curative	-,734750*	,146427	,001	-1,20010	-,26940
	Curative SCI	-,434500	,146427	,075	-,89985	,03085
Continus	Ctrl	,402750	,146427	,113	-,06260	,86810
	SCI	,532750*	,146427	,020	,06740	,99810
	Continus SCI	,398250	,146427	,119	-,06710	,86360
	Curative	-,202000	,146427	,738	-,66735	,26335
	Curative SCI	,098250	,146427	,983	-,36710	,56360
Continus SCI	Ctrl	,004500	,146427	1,000	-,46085	,46985
	SCI	,134500	,146427	,937	-,33085	,59985
	Continus	-,398250	,146427	,119	-,86360	,06710
	Curative	-,600250*	,146427	,007	-1,06560	-,13490
	Curative SCI	-,300000	,146427	,355	-,76535	,16535
Curative	Ctrl	,604750*	,146427	,007	,13940	1,07010
	SCI	,734750*	,146427	,001	,26940	1,20010
	Continus	,202000	,146427	,738	-,26335	,66735
	Continus SCI	,600250*	,146427	,007	,13490	1,06560
	Curative SCI	,300250	,146427	,354	-,16510	,76560
Curative SCI	Ctrl	,304500	,146427	,340	-,16085	,76985
	SCI	,434500	,146427	,075	-,03085	,89985
	Continus	-,098250	,146427	,983	-,56360	,36710
	Continus SCI	,300000	,146427	,355	-,16535	,76535
	Curative	-,300250	,146427	,354	-,76560	,16510

*. The mean difference is significant at the 0.05 level.

(c) <u>Wnt3 expression levels across different zebrafish larvae experimental</u>

groups

ANOVA

Wnt3

	Sum of Squares	df	Mean Square	F	Sig.
Between Groups	2,996	5	,599	21,975	,000
Within Groups	,491	18	,027		
Total	3,487	23			

Post Hoc Tests

Multiple Comparisons

Dependent Variable: Wnt3

Tukey HSD

(I) Group	(J) Group	Mean Difference (I-J)	Std. Error	Sig.	95% Confidence Interval	
					Lower Bound	Upper Bound
Ctrl	SCI	,300250	,116763	,155	-,07083	,67133
	Continus	-,600750*	,116763	,001	-,97183	-,22967
	Continus SCI	-,501000*	,116763	,005	-,87208	-,12992
	Curative	-,699550*	,116763	,000	-1,07063	-,32847
	Curative SCI	-,200975	,116763	,536	-,57205	,17010
SCI	Ctrl	-,300250	,116763	,155	-,67133	,07083
	Continus	-,901000*	,116763	,000	-1,27208	-,52992
	Continus SCI	-,801250*	,116763	,000	-1,17233	-,43017
	Curative	-,999800*	,116763	,000	-1,37088	-,62872
	Curative SCI	-,501225*	,116763	,005	-,87230	-,13015
Continus	Ctrl	,600750*	,116763	,001	,22967	,97183
	SCI	,901000*	,116763	,000	,52992	1,27208
	Continus SCI	,099750	,116763	,953	-,27133	,47083
	Curative	-,098800	,116763	,954	-,46988	,27228
	Curative SCI	,399775*	,116763	,031	,02870	,77085
Continus SCI	Ctrl	,501000*	,116763	,005	,12992	,87208
	SCI	,801250*	,116763	,000	,43017	1,17233
	Continus	-,099750	,116763	,953	-,47083	,27133
	Curative	-,198550	,116763	,548	-,56963	,17253
	Curative SCI	,300025	,116763	,156	-,07105	,67110
Curative	Ctrl	,699550*	,116763	,000	,32847	1,07063
	SCI	,999800*	,116763	,000	,62872	1,37088
	Continus	,098800	,116763	,954	-,27228	,46988
	Continus SCI	,198550	,116763	,548	-,17253	,56963
	Curative SCI	,498575*	,116763	,005	,12750	,86965
Curative SCI	Ctrl	,200975	,116763	,536	-,17010	,57205
	SCI	,501225*	,116763	,005	,13015	,87230

	Continus	-,399775*	,116763	,031	-,77085	-,02870
	Continus SCI	-,300025	,116763	,156	-,67110	,07105
	Curative	-,498575*	,116763	,005	-,86965	-,12750

*. The mean difference is significant at the 0.05 level.
